# P-203. Successful Elimination of *Clostridioides difficile* Admission Surveillance in favor of a Two Step Stool Algorithm and Early Detection Stool Counter

**DOI:** 10.1093/ofid/ofae631.407

**Published:** 2025-01-29

**Authors:** Donna Schora, Carolyn Hines, Cherie Faith Monsalud, Chethra Muthiah, Jennifer Grant, Maureen Kharasch

**Affiliations:** NorthShore University HealthSystem, Arlington Heights, Illinois; Endeavor Health, Evanston, Illinois; NorthShore University HealthSystem, Arlington Heights, Illinois; NorthShore University HealthSystem, Arlington Heights, Illinois; NorthShore University HealthSystem, Arlington Heights, Illinois; Endeavor Health, Evanston, Illinois

## Abstract

**Background:**

We began *Clostridioides difficile* (Cdif) admission surveillance (CDAS) in 2017 to decrease the rate of hospital onset (HO) Cdif across our hospital system. We created an algorithm to screen patients with a high risk of Cdif colonization and flag them by a best practice alert in the electronic medical record (EMR). The surveillance program helped to mitigate the spread of Cdif as evidenced by our HO Cdif rate the following 6 years. In 2023, we adopted a 2 step Cdif testing algorithm for symptomatic patients featuring a stool Cdif PCR test followed by EIA testing for PCR positives. This program reduced our hospital onset Cdif rate by 60% (unpublished data). We also reviewed lab test utilization and wondered if the admission screen test could be eliminated in favor of the 2 step system and an early detection stool counter. The goal of the trial was to determine if elimination of the admission testing can keep our Cdif HO rate at the level of our 2023 performance ( >75% of NHSN benchmark).

Number of Clostridioides difficile admission tests and Standardized Infection Ratio

**Figure:**
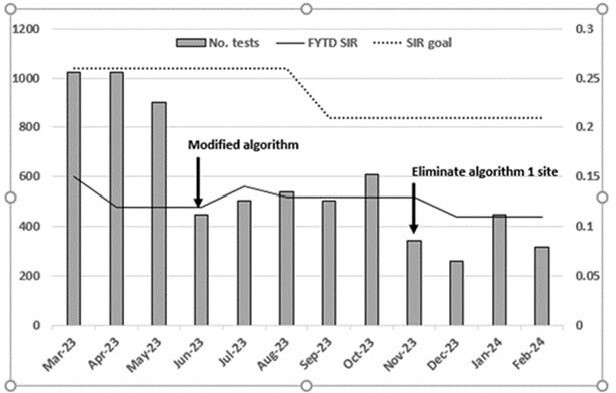

**Methods:**

The CDAS algorithm identifies hospitalized patients with prior nursing home or hospital admission, history of Cdif disease/colonization and a control group. A rectal sample was collected and tested with PCR. In June of 2023 the algorithm was updated to include ‘the receipt of antibiotics within the past 24 hours’.

The 2 step Cdif testing for symptomatic patients is a stool Cdif PCR test followed by EIA testing for PCR positives.

In November 2023, we stopped the admission testing at 1 of 4 hospitals, and in March 2024, testing stopped at all 4 hospitals.

A stool counter in the EMR tells the number of bowel movements in the first 48 hours of admission.

The standardized infection ratio (SIR) of hospital onset Cdif infection is used to measure the success of the trial.

**Results:**

Results of the trial are shown in Figure 1. The modified algorithm reduced the tests by half. The rate of HO Cdif infection has remained below the SIR goal for fiscal years 2023 (0.26) and 2024 (0.21). Estimated savings for calendar year 2023 was $210,000 as provided by the financial department.

**Conclusion:**

Replacing the Cdif admission surveillance with a 2 step test process for symptomatic patients did not affect the SIR for HO Cdif during the trial period. Eliminating the admission screen test will help to save financial resources while maintaining patient wellbeing.

**Disclosures:**

**All Authors**: No reported disclosures

